# Increase of angiotensin II type 1 receptor auto-antibodies in Huntington’s disease

**DOI:** 10.1186/1750-1326-9-49

**Published:** 2014-11-15

**Authors:** De-Hyung Lee, Harald Heidecke, Alexandra Schröder, Friedemann Paul, Rolf Wachter, Rainer Hoffmann, Gisa Ellrichmann, Duska Dragun, Anne Waschbisch, Johannes Stegbauer, Peter Klotz, Ralf Gold, Ralf Dechend, Dominik N Müller, Carsten Saft, Ralf A Linker

**Affiliations:** Department of Neurology, Friedrich Alexander University Erlangen-Nürnberg, Schwabachanlage 6, Erlangen, 91054 Germany; Department of Neurology, St. Josef Hospital, Ruhr University Bochum, Gudrunstr. 56, Bochum, 44791 Germany; CellTrend GmbH, Im Biotechnologiepark, Luckenwalde, 14943 Germany; NeuroCure Clinical Research Center and Clinical and Experimental Multiple Sclerosis Research Center, Department of Neurology, Charité-Universitätsmedizin Berlin, Berlin, 10117 Germany; Department of Cardiology, Georg-August University Göttingen, Göttingen, Germany; Department of Nephrology and Cardiovascular Research, Campus Virchow-Klinikum, Charité Universitätsmedizin Berlin, Berlin, Germany; Department of Nephrology, Medical Faculty, University Düsseldorf, Düsseldorf, Germany; Experimental and Clinical Research Center, a joint cooperation between the Charitè Medical, Faculty and the Max-Delbruck Center for Molecular Medicine, Berlin, Germany; Klinik und Poliklinik für Kardiologie und Nephrologie, Helios Klinikum Berlin-Buch, Berlin, Germany

**Keywords:** Angiotensin II type I receptor, Huntington’s disease, Multiple sclerosis, Neurodenegeration, Neuroinflammation

## Abstract

**Background:**

In the recent years, a role of the immune system in Huntington’s disease (HD) is increasingly recognized. Here we investigate the presence of T cell activating auto-antibodies against angiotensin II type 1 receptors (AT1R) in all stages of the disease as compared to healthy controls and patients suffering from multiple sclerosis (MS) as a prototype neurologic autoimmune disease.

**Results:**

As compared to controls, MS patients show higher titers of anti-AT1R antibodies, especially in individuals with active disease. In HD, anti-AT1R antibodies are more frequent than in healthy controls or even MS and occur in 37.9% of patients with relevant titers ≥ 20 U/ml. In a correlation analysis with clinical parameters, the presence of AT1R antibodies in the sera of HD individuals inversely correlated with the age of onset and positively with the disease burden score as well as with smoking and infection.

**Conclusions:**

These data suggest a dysfunction of the adaptive immune system in HD which may be triggered by different stimuli including autoimmune responses, infection and possibly also smoking.

## Background

Huntington’s disease (HD) is a devastating, progressive neurodegenerative disease with autosomal dominant inheritance, characterized by movement disorder, cognitive decline and behavioral abnormalities. It is caused by a trinucleotide CAG repeat expansion (≥36) in the gene encoding the protein huntingtin, localized on chromosome 4 [1]. Over the last two decades, knowledge on the pathophysiology and molecular biology of HD has significantly extended and the contribution of non-CNS tissues to pathogenesis and clinical symptomatology is increasingly recognized. Besides changes in the CNS, additional systemic abnormalities have been identified including endocrine dysfunction and immune activation [2, 3]. Neuroinflammatory pathomechanisms have been observed in several neurodegenerative diseases which may contribute to the cascade of events leading to neuronal degeneration [4–8]. In HD patients, activation of the peripheral immune system and in particular an up regulation of innate immune responses including microglia activation has been repeatedly reported [9–11]. Yet, only scarce data exist on the activation of adaptive immune responses in HD which may be characterized by an augmented T cell response or the presence of auto-antibodies. One such approach was the detection of anti-gliadin antibodies, which were detected in one study in 44.2% of HD patients [12].

Candidate auto-antibodies involved in dysfunction of the adaptive immune system are antibodies against angiotensin II type 1 receptors (AT1R). AT1R mediates the cellular effects of angiotensin II, the major effector molecule of the renin angiotensin aldosterone system (RAAS), which is a well-known regulator of salt homeostasis and blood pressure. Yet, there is also some pivotal evidence that angiotensin II and anti-AT1R antibodies play an important role in inflammatory processes. In particular, anti-AT1R antibodies may contribute to pre-eclampsia and are involved in acute transplant rejection and graft loss [13]. Further studies show that pre-transplant sensitization against AT1R increased the risk for acute rejection [14]. An antibody titer > 10 U/ml was determined as independent risk factor for rejection. In the autoimmune disease systemic sclerosis, anti-AT1R antibodies may serve as biomarker for risk assessment of disease progression, contribute to disease pathogenesis and predict disease related mortality [15]. So far, an association between anti-AT1R antibodies and neurodegenerative diseases has not been investigated. Here we analyze the presence of anti-AT1R antibodies in HD patients as compared to healthy controls and patients suffering from multiple sclerosis (MS) as a prototypic autoimmune disease associated with the production of distinct auto-antibodies [16, 17]. In HD, anti-AT1R antibodies are present at high titers.

## Results

### Detection of anti-AT1R antibodies in HD individuals

In 132 HD participants with genetically definite HD from all stages of the disease serum anti-AT1R antibodies were analysed via ELISA. The main demographic and clinical characteristics of HD subjects at the time point of investigation are reported in Table 1. 46 participants were smokers, 18 had an infection, 16 reported any kind of an allergy, 36 were on serotonin reuptake inhibitors as an antidepressant medication, 59 on an anti-dopaminergic drug and 78 took any kind of other medication.

**Figure 1:**
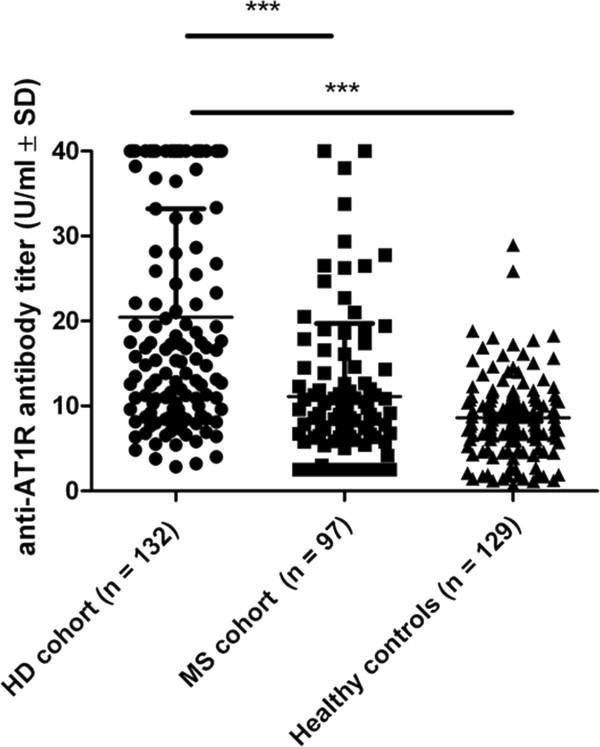
**Dot plot graph showing anti-AT1R antibody titers (U/ml) in HD individuals (n = 132), MS patients (n = 97) and healthy controls (n = 129).**

The mean anti-AT1 antibody titer in the HD cohort was determined as 20.5 ± 12.8 U/ml which was significantly higher than in healthy controls (mean titer: 8.6 ± 4.9 U/ml, Figure 1). Upon analysis of ranks close to quartiles 23.8% of values were < 10 U/ml, 23.5% between 10–16 U/ml, 25.8% between 16–35 U/ml and 25% ≥ 35 U/ml. Assuming values > 15 U/ml as relevant titers, 71 of 132 patients (53.8%) display relevant anti-AT1R antibodies. Assuming 20 U/ml as relevant cutoff, 50 of 132 HD individuals (37.9%) displayed relevant anti-AT1R serum antibodies. A total of 29 patients (21.9%) showed titers reaching the ceiling value > 40 U/ml. There were no significant titer differences between males and females.

**Table 1:** **Baseline data of HD individuals and healthy controls**

Parameter	HD individuals	Healthy controls
	(n = 132)	(n = 129)
Age [yr]	46.9 ± 12.8	47.5 ± 9.8
	(21–89)	(22–56)
Weight [kg]	68.4 ± 14.3	73.4 ± 14
	(40–101)	(50–115, n = 103)
Height	171.7 ± 9.1	170 ± 9.7
	(157–197)	(150–191, n = 103)
Smoking [%]	34.8	34.4 (n = 125)
CAG expanded	44.7 ± 4.7	-----
	(39–70)	
Disease burden score	403.9 ± 133.6	-----
	(91–825)	
Onset motor [yr]	41.5 ± 12.1	-----
	(10–72, n = 102)	
Onset psychiatric [yr]	42.2 ± 12.1	-----
	(15–73, n = 57)	
Duration of disease [yr]	7.6 ± 5.1	-----
	(0.1-23, n = 102)	
YTO Langbehn [yr]	16.9 ± 9.1	-----
	(5–43, n = 30)	
UHDRS MS	41.9 ± 29.7	-----
	(0–96)	
UHDRS TFC	7.6 ± 4.5	-----
	(0–13)	
UHDRS IS	70.5 ± 26.5	-----
	(10–100)	
UHDRS CS	159.5 ± 108.8	-----
	(0–379)	
Tapping dominant	162 ± 43.7	-----
	(65–233, n = 55)	
Tapping non-dominant	139.1 ± 46.9	-----
	(40–207, n = 55)	
Peg board dominant [sec]	52.6 ± 13.6	-----
	(32.5-88.5, n = 55)	
Peg board non-dominant [sec]	58.6 ± 16.0	-----
	(38.7-100, n = 55)

In a selected cohort of nine patients, follow-up samples were collected one year later. Upon follow-up anti-AT1R antibody titers were surprisingly stable and only differed by a mean of 3.7 ± 4.3 U/ml (p = not significant). Relevant titer changes (assuming the cutoff of 20 U/ml) occurred only in 2/9 (22.2%) cases.

To further assess the significance of our finding in HD individuals, we additionally analyzed the presence of anti-AT1R antibodies in a cohort of a total of 97 MS patients (baseline data given in Table 2). In the total cohort, 20/97 (20.6%) patients displayed titers > 15 U/ml, 13/97 (13.4%) displayed titers > 20 U/ml and 2/97 (2.1%) showed titers reaching the ceiling value of > 40 U/ml. The mean titer was 11.1 ± 8.6 U/ml which was significantly lower than in HD individuals (Figure 1, p < 0.001). In the total MS cohort, antibody titers did not correlate with disease duration, disease course (RR-MS vs. SP-MS), disease severity as measured by EDSS or immunomodulatory treatment. However, MS patients with active disease defined as relapse or MRI activity in the previous 3 months displayed higher antibody titers than patients with stable relapsing-remitting or progressive disease (mean 25.6 ± 11.8 versus 12.0 ± 7.1 U/ml, n = 9 versus 89; p = 0.0005).

**Table 2:** **Baseline data of MS patients, n = 97**

Parameter	MS individuals
Mean Age (years ± SD)	39.3 ± 10.2
Gender Male/Female (%)	40.2/59.8%
Disease Course RR-MS/SP-MS (%)	80.4/19.6%
Disease Duration (years ± SD)	7.0 ± 5.4
Median EDSS (range)	2.5 (0–8.5)

### Correlation of anti-AT1R antibodies with clinical symptoms in HD

In a next step, we correlated absolute antibody values with clinical features of our HD cohort. Parameters included baseline information (age, gender, height, body weight), medication (neuroleptics, SSRI, other), information of disease onset (onset of motor symptoms, onset of psychiatric symptoms), scales for clinical disease stage (UHDRS, independency scale (IS), total functional capacity scale (TFC), disease duration and CAG repeats, measures of neuropsychology (CS) and motor function (MS) including more quantitative motor measures like tapping or peg board results and finally infection, smoking and allergy (Table 3). Antibody titers significantly correlated with motor onset of disease (r = -0.23; p = 0.02), duration of disease (r = 0.18, p = 0.035), UHDRS-MS (r = 0.22, p = 0.012), TFC (r = -0.18, p = 0.03) and IS (r = -0.21, p = 0.016) as well as disease burden score (r = 0.18, p = 0.04). The higher the antibody titer, the earlier was the onset of disease. Yet, antibody titers also significantly correlated with smoking (r = -0.26, p = 0.005) and the presence of infection at sampling (r = 0.27, p = 0.002), but not with allergies. Similar results were obtained when correlating these clinical features with quartiles of anti-AT1R antibodies or when assuming 15 U/ml as cutoff for positive titers (data not shown). In contrast, antibody titers did not correlate with smoking in the healthy control group (r = 0.022, p = 0.76).

**Table 3:** **Correlation of anti-AT1R antibodies with clinical features of HD individuals**

	R-value	P-value
Age	- 0.03	0.732
Gender	0.119	0.175
Height	0.044	0.646
Body weight	0.009	0.925
**Onset motor**	**- 0.23**	0.02*
Onset psychiatric	-0.23	0.095
**Duration of disease**	**0.18**	0.035*
**UHDRS**	**0.22**	0.012*
**TFC**	**- 0.18**	0.03*
**IS**	**-0.21**	0.016*
CAG not-expanded	0.02	0.822
CAG expanded	0.155	0.076
**CAG Index**	**0.18**	0.04*
Year to onset (YTO) Langbehn	0.092	0.627
Shoulson scale	0.15	0.087
Neuroleptic Intake	0.149	0.088
SSRI intake	-0.63	0.473
Other Medication	0.011	0.897
**Smoking**	**-0.26**	0.005**
**Infection**	**0.27**	0.002**
Allergy	0.097	0.268
Tapping dominant	-0.01	0.921
Tapping non-dominant	0.0	0.998
Peg board dominant	0.001	0.992
Peg board non-dominant	0.088	0.992

Significant correlations are shown in bold, asterisks denote significance with *p < 0.05, **p < 0.001.

After exclusion of HD patients with more severe stages of the disease (Shoulson stage 4 and 5; n = 107 remaining), there were no significant correlations of absolute antibody titers with clinical data. Yet in this very subgroup of patients, the presence of anti-AT1R antibodies (cutoff 20 U/ml) did correlate with motor onset of disease (r = -0.28; p = 0.016) and psychiatric onset of disease (r = -0.36; p = 0.026).

In contrast, anti-AT1R antibodies did not correlate with age, gender, body height or body weight, medication, motor or neuropsychological function, CAG repeat length, Shoulson scale, years to onset or disease duration in HD individuals.

After numerical calculation of antibody titers >40 to obtain exact values (n = 132, mean 32.7 ± 37.4; range: 2.9 to 146.5), titers did not correlate with motor age of onset any more, but we now found stronger correlations with duration of disease (r = 0.244, p = 0.005), UHDRS-MS (r = 0.367, p < 0.001), TFC (r = -0.344, p < 0.001) and IS (r = -0.415, p < 0.001) as well as disease burden score (r = 0.302, p = 0.001). In addition, there was a significant correlation with cognitive sum score (r = -0.293, p = 0.001), and expanded CAG (r = 0.193, p = 0.027). Antibody titers still significantly correlated with smoking (r = -0.264, p = 0.004) and the presence of infection at sampling (r = 0.315, p < 0.001), but not with allergies.

### Modeling the relevance of anti-AT1R antibodies in HD

In regression analyses, we modeled the prediction of antibody titers by smoking and infection versus HD related variables in step wise models. Smoking as well as infection were significant predictors for the presence of anti-AT1R antibodies and explained 15% of the variance (r^2^ = 0.151). When including the HD relevant variables independence scale (IS), CAG expanded and Shoulson scale as well as “motor” or “psychiatric onset” of disease to the model, IS alone explains 25.8%, IS and CAG expanded together explain 34.8% and all variables explain 41.5% of the variance (r^2^ = 0.415). A final stepwise model included independence scale (IS) as predictor and as next steps the variables “onset motor” and smoking. IS alone explains 14.5% of the variance, IS and onset motor together explain 19.2% of the variance, IS and “onset motor” and smoking together explain 23.7% of the variance (Table 4).

**Table 4:** **Modelling the relation of anti-AT1R antibodies in HD individuals to disease-related outcomes and smoking as well as infection**

		Standardized Beta	T	p-Value
Model - Step 1	IS	- 0.50	- 4.0	< 0.0001
Model - Step 2	IS	0.50	- 4.3	< 0.0001
	CAG expanded	0.30	2.5	0.016
Model - Step 3	IS	1.1	- 3.9	< 0.0001
	CAG expanded	0.30	2.6	0.014
	Shoulson	0.60	- 2.2	0.03

A linear regression was performed to predict anti-AT1R antibody titers via infection, smoking and HD-relevant variables with additional variables “onset motor” and “onset psychiatric” (n = 48, i.e. only smokers with infection and motor as well as psychiatric onset of disease were included). A stepwise analysis revealed in a first step independence scale (IS), in a second step CAG expanded and in a third step the variable Shoulson scale as predictor. Other variables were not relevant for predicting the presence of anti-AT1R antibody titers in this subgroup.

A covariate analysis of HD patients suffering from psychiatric symptoms (“psychiatric onset”) showed that only the presence of anti-AT1R antibodies (cutoff 20 U/ml), but not smoking or infection was a significant covariate. Further covariate analyses of the variables disease burden score, duration of disease or Shoulson scale after grouping according to the presence of antibody titers and smoking as well as infection as co-variates revealed infection as significant covariate in all analyses.

Upon exclusion of smokers and participants with infection from the HD cohort, a total of 55 individuals remained left for further analyses. The mean anti-AT1R antibody titer in this group was 21.1 ± 12.1 U/ml which was still significantly higher than in the MS cohort and in healthy controls (each p < 0.0001). Assuming an antibody titer of 20 U/ml as cutoff, statistical analyses by Mann–Whitney testing confirmed significant differences between patients with or without significant anti-AT1R antibody titers for “onset motor” (p = 0.046) and “onset psychiatric” (p = 0.044). For the variables “onset” and “years to onset”, there was a trend in this cohort towards earlier time points in the antibody positive subgroup (n = 55, p = 0.096).

## Discussion

In this study, we found that anti-AT1R antibodies are present in HD individuals and MS patients at high titers. The positive detection of anti-AT1R antibodies in the sera of MS patients was associated with recent disease activity. In HD individuals, anti-AT1R antibody titers inversely correlated with the age of onset and were also linked to smoking and infection.

The presence of anti-AT1R IgG antibodies in HD implies an increased activity of the adaptive immune system and in particular B cell and plasma cell activity in HD patients. Yet, there are no data on a general increased IgG production or a general increase of auto-antibodies in HD patients. So far, the only auto-antibody described in HD is the detection of anti-gliadin antibodies in 44.2% of HD patients [12]. Here, further studies on subsets of the B cells lineage and antibody profiles in HD are clearly of interest.

MS patients display higher anti-AT1R IgG antibodies than healthy controls. The finding of anti-AT1R antibodies in MS adds this antibody to a range of different auto-antibodies in MS directed against myelin and glial proteins, most recently e.g. the Kir4.1 potassium channel [16–19]. Although these antibody may play a pathophysiological role, MS is generally viewed as a primarily T cell mediated autoimmune disease. The concept of anti-AT1R antibodies as drivers of increased T cell responses was already proven in renal transplant rejection and rheumatologic disease [13, 20]. Thus the finding of anti-AT1R antibodies with particularly high titers in patients with active disease is well in line with the pathophysiological role of these auto-antibodies in other diseases and also with the presumed pathomechanisms of MS [21]. The role of anti-AT1R antibodies as a new marker of disease activity in relapsing-remitting MS deserves further investigation in longitudinal studies.

The presence of anti-AT1R antibodies in HD also argues for an increased activation of T cells in HD patients which harbor AT1R. In autoimmune disease (see above), the role of T cells is much better established than in HD which has essentially been characterized as a genetically mediated neurodegenerative disease. Yet, a role for the innate immune system in HD is increasingly recognized and immune factors may constitute modifiers of the disease. So far, data on the adaptive immune response in HD only refer to some studies on cytokines like interleukin-4 or lymphocyte numbers. However, the significance of T cells to the pathophysiology of the disease is still largely unclear. Our findings in HD further add to the notion of an interlocking of inflammatory and possibly also neuroinflammatory processes on the one hand and neurodegenerative processes on the other hand. Our observations well match with recent reports contributing to an increasingly recognized role of the immune system in HD [22, 23]. Indeed, an over-active adaptive immune response in the peripheral blood as indicated by or even driven by the presence of anti-AT1R antibodies may hasten the onset of disease and thus contribute to neurodegeneration. Further studies on the role of on the role of the adaptive immune system and in particular effects of anti-AT1R antibodies on T cell function in HD and also other neurodegenerative diseases are warranted. Indeed, immune factors like anti-AT1R antibodies may add to the determinants influencing age of onset and rate of progression in HD which were previously discussed [24, 25].

It is tempting to speculate that auto-antibody production and dysfunction of the adaptive immune system in HD may be directly triggered by huntingtin aggregates and mitochondrial dysfunction in immune cells. This process may mimic a premature ageing of the immune system as the disease progresses. Mitochondrial dysfunction is a well-known part in the pathophysiology of HD and may lead to oxidative stress [26–30]. In immune cells, oxidative stress is discussed to induce inflammasomes and mitochondrial dynamics are known to influence T cell function [31]. A direct link between inflammatory processes and mitochondrial dysfunction in T cells is also supported by severe ultra-structural mitochondrial changes in lymphoblasts from homozygous HD patients [32]. Interestingly, there is a correlation between anti-AT1R antibody titer and onset of HD, but no correlation between anti-AT1R titer and years to onset. These data may argue for a role of anti-AT1R not in the pre-symptomatic phase, but rather during the course of the manifest disease. This observation may additionally speak for the HD related specificity of our findings and against a pure association with (over the course of the disease unchanged) life style factors (see below).

In our study, the presence of anti-AT1R antibodies also correlates with smoking and infection in HD individuals. Statistical modelling argues for some smoking and infection independent effects of HD and anti-AT1R antibody titers still remain significantly higher after exclusion of smokers and individuals with infections were excluded. The specificity of these data is further strengthened by the lack of a similar association in healthy individuals. Possibly, smoking may serve as a trigger in a susceptible population for the generation of anti-AT1R auto-antibodies at a certain disease stage. Thus, it is easily conceivable that infection or subclinical airway inflammation induced by smoking may directly or indirectly contribute to the induction of auto-antibodies. In addition, it cannot be excluded that also ageing may contribute to the generation of anti-AT1R antibodies in humans. Yet, our control cohort is well-matched to HD individuals thus rather excluding a confounding factor. In addition, previous studies on anti-AT1R antibodies did not describe an increased frequency of this auto-antibody with age alone and the presence of significant anti-AT1R antibody titers in HD individuals did not correlate with age in our cohort.

In view of the presence of anti-AT1R antibodies in different autoimmune and cardiovascular diseases and HD, this antibody is obviously not disease-specific, but rather indicates a general, but disease-related mechanism for dysfunction of the adaptive immune system which may be triggered by different stimuli including autoimmune responses, infection and possibly also smoking.

In synopsis of the relation of anti-AT1R antibodies with smoking and onset of HD symptoms, it is tempting to speculate that life style factors such as smoking may directly influence the course of the disease via effects on the immune system. Indeed, the lung as organ affected by smoking has recently been implicated as an important regulator in T cell function [33]. In HD, anti-AT1R antibodies may provide a direct molecular link connecting smoking, an increased activation of the immune system and neurodegeneration. Similar associations between life style modifiers and progression of neurologic disease have recently been investigated for smoking and MS and caffeine intake and age at onset in HD [34, 35].

In summary, we show the presence of anti-AT1R antibodies in HD with a higher frequency than in MS patients and in healthy controls. Anti-AT1R antibodies correlated with disease activity in MS and with onset of disease and disease burden scores in HD. Future studies in HD and MS also including transfer experiments in animal models are needed to determine the exact pathogenic role of this antibody response.

## Material and methods

### HD subjects

HD subjects were recruited at the Department of Neurology, Ruhr University of Bochum. 132 genetically confirmed HD mutation carriers (72 (54.5%) women and 60 (45.4%) men; mean age 46.9 ± 12.8 years) were screened for anti-AT1R antibodies. 30 of the 132 HD mutation carriers were classified as pre-manifest (preHD), based on expert raters’ assessments of motor signs which were not sufficient for the diagnosis of HD (Diagnostic Confidence Level [DCL], item 17 of the UHDRS Motor Assessment) [36]. Sixteen participants of the 102 manifest HD participants were in Shoulson stage I, 35 in stage II, 26 in stage III, seventeen in stage IV and eight in Shoulson stage V, respectively (see also Table 1) [37]. In a subgroup of nine patients, follow-up was after 12 months. At each assessment, the following data and scores were determined: age, gender, weight, height and smoking behavior as well as scores on the motor (MSc), cognitive (verbal fluency test, symbol digit modalities test, Stroop color, Stroop word and Stroop interference, summarized as cognitive score (CS)), and independence (IS), as well as functional sections (TFC) of the validated Unified Huntington’s Disease rating scale (UHDRS) [36]. If possible, fine motor skills were measured by simple (tapping; higher motor impairment leads to lower test results) or complex (pegboard; higher motor impairment leads to higher test results) quantitative movement tests [38, 39]. Years to disease onset (YTO) for the preHD subjects were calculated by subtracting the subject’s age at the time of investigation from his or her estimated onset age using Langbehn’s formula [40]. Moreover we calculated the disease burden score (DBS = [CAG repeat - 35.5] × age) for each subject [41]. Additionally, patients were interviewed about medical problems including allergy, severe other diseases, medication intake and the presence of infections. Infections were excluded clinically for outpatients and excluded or confirmed clinically and by determination of the C-reactive protein for inpatients (all cases with Shoulson stage III to V) and all cases clinically suspicious for an infection. Patients with severe other diseases, such as a carcinoma, were excluded.

### MS patients and healthy controls

A cohort of MS patients from Bochum or NeuroCure Center, Berlin, Charité was enrolled in this study. Diagnosis was confirmed by the treating neurologist based on the revised 2010 McDonald clinical and radiologic criteria for MS [42]. 59.8% of MS patients were female and 40.2% male, mean age was 39.3 ± 10.2 years, 80.4% had relapsing remitting (RR-MS) and 19.6% had secondary progressive MS (SP-MS, see Table 2). 69.1% of patients received a disease modifying therapy with injectables (beta interferon or glatiramer acetate), 15.5% were without treatment, 12.3% received a second line therapy (natalizumab, mitoxantrone) and 3.1% were on other treatments. Data from MS patients were correlated with clinical course, disease modifying treatment, relapse and MRI activity and disability (expanded disability score scale, EDSS). Active disease was defined as relapse or MRI activity in the last 3 months prior to serum sampling.

Healthy controls were recruited at the University Hospitals of Goettingen and Erlangen after obtaining informed consent. 70.5% of control individuals were female and 29.5% male. Controls were matched to HD subjects for age (47.5 ± 9.8 years) and smoking status (34.4% smokers in the control group vs 34.8% in the HD group, see also Table 1.

### Solid-phase enzyme linked immunosorbent assay

Written informed consent to use serum samples for research purposes was obtained from each individual. Samples were collected in a standardized manner and were analyzed for antibodies against the AT1R. A commercially available sandwich enzyme linked immunosorbent assay (ELISA; CellTrend GmbH, Luckenwalde, Germany) to measure anti-AT1R autoantibodies was employed as described previously [13–15]. Briefly, microtiter 96-well polystyrene plates were coated with extracts from Chinese hamster ovary cells overexpressing the human AT1R. To maintain the conformational epitopes of both receptors, 1 mM calcium chloride was added to each buffer. Duplicate samples of a 1:100 serum dilution were incubated at 4°C for 2 h. After washing steps, plates were incubated for 60 min with a 1:20000 dilution of horseradish peroxidase-labelled goat anti-human IgG (Jackson, West Grove, Pennsylvania, USA) used for detection. In order to obtain a standard curve, plates were incubated with a ready-to use standard at 2.5, 5, 10, 20, and 40 U/ml. The assay was validated as compared to a cardiomyocyte bioassay (for further assay details see also Dragun) [43]. The ELISA was validated according to the Food and Drug Administration’s ‘Guidance for Industry: Bioanalytical Method Validation’. The inter-assay variability was 7% and the intra-assay variability was 6%. Persons who were unaware of the patients’ characteristics performed the assays. Values reaching the ceiling of > 40 U/l were counted as “40”, values equal to or below the detection limit of 2.5 U/l were counted as “2.5”. In post-hoc analyses, numerical calculations were performed to obtain exact values for titers reaching the ceiling value > 40 U/ml.

### Ethics

The study was approved by the local ethics committees in Erlangen, Goettingen, Bochum and Berlin (Nr 3184–08 Ruhr-University Bochum and ZS EK 13 255/07 Berlin) and was conducted in accordance to the Declaration of Helsinki in its currently applicable form, the guidelines of the International Conference on Harmonization of Good Clinical Practice (ICH-GCP) and the applicable German laws. Participants or their legal guardians gave written informed consent.

### Statistical analysis

Statistical analysis of baseline data and comparison between groups was performed using *chi-square*-, Mann–Whitney *U*- and Kruskal Wallis tests calculated via PRISMS (GraphPad Software, Inc., La Jolla, CA, USA). Testing of probability distributions in our samples was performed using Kolmogorow-Smirnov-test. The association between anti-AT1R antibodies of each participant and type of onset, smoking (defined as regular nicotine consumption ever day; yes/no), infection (yes/no), UHDRS-MS, IS, TFS, CS and DBS was tested by correlation and linear regression models controlling for anti-AT1R antibody titers. For correlation analysis, Spearman-Rho analysis was used for non-parametric data and Pearson analysis if data showed normal distribution. Testing for significance was achieved by using Wald- *chi-square*-test. Correlation analysis and linear regression were computed using PASW-Statistics 18 (IBM SPSS, NY, USA). Significance was accorded to *p*-values *p < .05; **p < .01; ***p < .001.

## Authors’ information

Carsten Saft and Ralf A Linker share senior authorship.

## Abbreviations

AT1R: Angiotensin II type 1 receptors
CNS: Central nerve systems
DBS: Disease burden score
ELISA: Enzyme linked immunosorbent assay
HD: Huntington’s disease
MS: Multiple sclerosis
MRI: Magnetic resonance imaging
RAAS: Renin angiotensin aldosterone system
RRMS: Relapsing remitting multiple sclerosis
SPMS: Secondary progressive multiple sclerosis
SSRI: Selective serotonin reuptake inhibitors
TFC: Total functional capacity scale.

## References

[1] The Huntington’s Disease Collaborative Research Group (1993). A novel gene containing a trinucleotide repeat that is expanded and unstable on Huntington’s disease chromosomes. Cell.

[2] Bjorkqvist M, Petersen A, Bacos K, Isaacs J, Norlén P, Gil J, Popovic N, Sundler F, Bates GP, Tabrizi SJ, Brundin P, Mulder H (2006). Progressive alterations in the hypothalamic-pituitary-adrenal axis in the R6/2 transgenic mouse model of Huntington’s disease. Hum Mol Genet.

[3] Leblhuber F, Walli J, Jellinger K, Tilz GP, Widner B, Laccone F, Fuchs D (1998). Activated immune system in patients with Huntington’s disease. Clin Chem Lab.

[4] Ellrichmann G, Petrasch-Parwez E, Lee DH, Reick C, Arning L, Saft C, Gold R, Linker RA (2011). Efficacy of fumaric acid esters in the R6/2 and YAC128 models of Huntington’s disease. PLoS One.

[5] Harry GJ, Kraft AD (2008). Neuroinflammation and microglia: considerations and approaches for neurotoxicity assessment. Expert Opin Drug Metab Toxicol.

[6] Hirsch EC, Hunot S (2009). Neuroinflammation in Parkinson’s disease: a target for neuroprotection?. Lancet Neurol.

[7] Lobsiger CS, Boillee S, Pozniak C, Khan AM, McAlonis-Downes M, Lewcock JW, Cleveland DW (2013). C1q induction and global complement pathway activation do not contribute to ALS toxicity in mutant SOD1 mice. Proc Natl Acad Sci U S A.

[8] Rogers J (2008). The inflammatory response in Alzheimer’s disease. J Periodontol.

[9] Bjorkqvist M, Wild EJ, Thiele J, Andre R, Lahiri N, Raibon E, Lee RV, Benn CL, Soulet D, Magnusson A, Woodman B, Landles C, Pouladi MA, Hayden MR, Khalili-Shirazi A, Lowdell MW, Brundin P, Bates GP, Leavitt BR, Möller T, Tabrizi SJ (2008). A novel pathogenic pathway of immune activation detectable before clinical onset in Huntington’s disease. J Exp Med.

[10] Pavese N, Gerhard A, Tai YF, Ho AK, Turkheimer F, Barker RA, Brooks DJ, Piccini P (2006). Microglial activation correlates with severity in Huntington disease: a clinical and PET study. Neurology.

[11] Tai YF, Pavese N, Gerhard A, Tabrizi SJ, Barker RA, Brooks DJ, Piccini P (2007). Microglial activation in presymptomatic Huntington’s disease gene carriers. Brain.

[12] Bushara KO, Nance M, Gomez CM (2004). Antigliadin antibodies in Huntington’s disease. Neurology.

[13] Dragun D, Muller DN, Brasen JH, Fritsche L, Nieminen-Kelhä M, Dechend R, Kintscher U, Rudolph B, Hoebeke J, Eckert D, Mazak I, Plehm R, Schönemann C, Unger T, Budde K, Neumayer HH, Luft FC, Wallukat G (2005). Angiotensin II type 1-receptor activating antibodies in renal-allograft rejection. N Eng J Med.

[14] Giral M, Foucher Y, Dufay A, Van Huyen JP, Renaudin K, Moreau A, Philippe A, Hegner B, Dechend R, Heidecke H, Brouard S, Cesbron A, Castagnet S, Devys A, Soulillou JP, Dragun D (2013). Pretransplant sensitization against angiotensin II type 1 receptor is a risk factor for acute rejection and graft loss. Am J Transplant.

[15] Riemekasten G, Philippe A, Nather M, Slowinski T, Müller DN, Heidecke H, Matucci-Cerinic M, Czirják L, Lukitsch I, Becker M, Kill A, van Laar JM, Catar R, Luft FC, Burmester GR, Hegner B, Dragun D (2011). Involvement of functional autoantibodies against vascular receptors in systemic sclerosis. Ann Rheum Dis.

[16] Kraus V, Srivastava R, Kalluri SR, Seidel U, Schuelke M, Schimmel M, Rostasy K, Leiz S, Hosie S, Grummel V, Hemmer B (2014). Potassium channel KIR4.1-specific antibodies in children with acquired demyelinating CNS disease. Neurology.

[17] Srivastava R, Aslam M, Kalluri SR, Schirmer L, Buck D, Tackenberg B, Rothhammer V, Chan A, Gold R, Berthele A, Bennett JL, Korn T, Hemmer B (2012). Potassium channel KIR4.1 as an immune target in multiple sclerosis. N Eng J Med.

[18] Menge T, Lalive PH, von Budingen HC, Genain CP (2011). Conformational epitopes of myelin oligodendrocyte glycoprotein are targets of potentially pathogenic antibody responses in multiple sclerosis. J Neuroinflammation.

[19] Kuhle J, Pohl C, Mehling M, Kuhle J, Pohl C, Mehling M, Edan G, Freedman MS, Hartung HP, Polman CH, Miller DH, Montalban X, Barkhof F, Bauer L, Dahms S, Lindberg R, Kappos L, Sandbrink R (2007). Lack of association between antimyelin antibodies and progression to multiple sclerosis. N Eng J Med.

[20] Silveira KD, Coelho FM, Vieira AT, Barroso LC, Queiroz-Junior CM, Costa VV, Sousa LF, Oliveira ML, Bader M, Silva TA, Santos RA, Silva AC, Teixeira MM (2013). Mechanisms of the anti-inflammatory actions of the angiotensin type 1 receptor antagonist losartan in experimental models of arthritis. Peptides.

[21] Frohman EM, Racke MK, Raine CS (2006). Multiple sclerosis–the plaque and its pathogenesis. N Eng J Med.

[22] Crotti A, Benner C, Kerman BE, Gosselin D, Lagier-Tourenne C, Zuccato C, Cattaneo E, Gage FH, Cleveland DW, Glass CK (2014). Mutant Huntingtin promotes autonomous microglia activation via myeloid lineage-determining factors. Nat Neurosci.

[23] Hsiao HY, Chiu FL, Chen CM, Wu YR, Chen HM, Chen YC, Kuo HC, Chern Y (2014). Inhibition of soluble tumor necrosis factor is therapeutic in Huntington’s disease. Hum Mol Genet.

[24] Buruma OJ, Van der Kamp W, Barendswaard EC, Roos RA, Kromhout D, Van der Velde EA (1987). Which factors influence age at onset and rate of progression in Huntington’s disease?. J Neurol Sci.

[25] Myers RH, Sax DS, Koroshetz WJ, Mastromauro C, Cupples LA, Kiely DK, Pettengill FK, Bird ED (1991). Factors associated with slow progression in Huntington’s disease. Arch Neurol.

[26] Arning L, Haghikia A, Taherzadeh-Fard E, Saft C, Andrich J, Pula B, Höxtermann S, Wieczorek S, Akkad DA, Perrech M, Gold R, Epplen JT, Chan A (2010). Mitochondrial haplogroup H correlates with ATP levels and age at onset in Huntington disease. J Mol Med.

[27] Bossy-Wetzel E, Petrilli A, Knott AB (2008). Mutant huntingtin and mitochondrial dysfunction. Trends Neurosci.

[28] Saft C, Zange J, Andrich J, Müller K, Lindenberg K, Landwehrmeyer B, Vorgerd M, Kraus PH, Przuntek H, Schöls L (2005). Mitochondrial impairment in patients and asymptomatic mutation carriers of Huntington’s disease. Mov Disord.

[29] Stuwe SH, Goetze O, Lukas C, Klotz P, Hoffmann R, Banasch M, Orth M, Schmidt WE, Gold R, Saft C (2013). Hepatic mitochondrial dysfunction in manifest and premanifest Huntington disease. Neurology.

[30] Taherzadeh-Fard E, Saft C, Andrich J, Wieczorek S, Arning L (2009). PGC-1alpha as modifier of onset age in Huntington disease. Mol Neurodegener.

[31] Quintana A, Hoth M (2012). Mitochondrial dynamics and their impact on T cell function. Cell Calcium.

[32] Squitieri F, Cannella M, Sgarbi G, Maglione V, Falleni A, Lenzi P, Baracca A, Cislaghi G, Saft C, Ragona G, Russo MA, Thompson LM, Solaini G, Fornai F (2006). Severe ultrastructural mitochondrial changes in lymphoblasts homozygous for Huntington disease mutation. Mech Ageing Dev.

[33] Odoardi F, Sie C, Streyl K, Ulaganathan VK, Schläger C, Lodygin D, Heckelsmiller K, Nietfeld W, Ellwart J, Klinkert WE, Lottaz C, Nosov M, Brinkmann V, Spang R, Lehrach H, Vingron M, Wekerle H, Flügel-Koch C, Flügel A (2012). T cells become licensed in the lung to enter the central nervous system. Nature.

[34] Manouchehrinia A, Tench CR, Maxted J, Bibani RH, Britton J, Constantinescu CS (2013). Tobacco smoking and disability progression in multiple sclerosis: United Kingdom cohort study. Brain.

[35] Simonin C, Duru C, Salleron J, Hincker P, Charles P, Delval A, Youssov K, Burnouf S, Azulay JP, Verny C, Scherer C, Tranchant C, Goizet C, Debruxelles S, Defebvre L, Sablonnière B, Romon-Rousseaux M, Buée L, Destée A, Godefroy O, Dürr A, Landwehrmeyer B, Bachoud-Levi AC, Richard F, Blum D, Krystkowiak P, REGISTRY Study of the European Huntington’s Disease Network, Huntington French Speaking Network (2013). Association between caffeine intake and age at onset in Huntington’s disease. Neurobiol Dis.

[36] Huntington Study Group (1996). Unified Huntington’s Disease Rating Scale: reliability and consistency. Mov Dis.

[37] Shoulson I (1981). Huntington disease: functional capacities in patients treated with neuroleptic and antidepressant drugs. Neurology.

[38] Saft C, Andrich J, Meisel NM, Przuntek H, Muller T (2003). Assessment of complex movements reflects dysfunction in Huntington’s disease. J Neurol.

[39] Saft C, Andrich J, Meisel NM, Przuntek H, Muller T (2006). Assessment of simple movements reflects impairment in Huntington’s disease. Mov Disord.

[40] Langbehn DR, Brinkman RR, Falush D, Paulsen JS, Hayden MR (2004). A new model for prediction of the age of onset and penetrance for Huntington’s disease based on CAG length. Clin Genet.

[41] Penney JB, Vonsattel JP, MacDonald ME, Gusella JF, Myers RH (1997). CAG repeat number governs the development rate of pathology in Huntington’s disease. Ann Neurol.

[42] Polman CH, Reingold SC, Banwell B, Clanet M, Cohen JA, Filippi M, Fujihara K, Havrdova E, Hutchinson M, Kappos L, Lublin FD, Montalban X, O’Connor P, Sandberg-Wollheim M, Thompson AJ, Waubant E, Weinshenker B, Wolinsky JS (2011). Diagnostic criteria for multiple sclerosis: 2010 revisions to the McDonald criteria. Ann Neurol.

[43] Dragun D (2013). The detection of antibodies to the Angiotensin II-type 1 receptor in transplantation. Methods Mol Biol.

